# Purr-ceiving feelings: domestic cats respond to intraspecific cues of emotion

**DOI:** 10.7717/peerj.21292

**Published:** 2026-05-25

**Authors:** Kiki E. Spoelstra, Jennifer Vonk, Evy van Berlo, José Kok, Mariska E. Kret

**Affiliations:** 1 Institute of Psychology, Cognitive Psychology Unit, Leiden University, Leiden, Netherlands; 2 Ouwehand Zoo, Rhenen, Netherlands; 3 Department of Psychology, Oakland University, Rochester, Michigan, United States; 4 Department of Biology, Animal Behaviour and Cognition, Utrecht University, Utrecht, Netherlands

**Keywords:** Emotion perception, Social cognition, *Felis silvestris* *catus*, Attention, Cat communication

## Abstract

**Background:**

Successful interpretation of emotion expressions is a critical aspect of communication in both solitary and group-living species, although it is seldom investigated in the former. Because of their flexible sociality, domestic cats (*Felis silvestris* *catus*) are an interesting species within which to study intraspecific emotion perception.

**Methods:**

Adopting a multidimensional approach, we presented cats (*n* = 24) with visual and auditory stimuli that represented two levels of valence and activity, which served as a proxy for arousal. Thus, each cat experienced four auditory and four visual stimulus presentations lasting 2 min each in which we measured the cats’ gaze duration and frequency, proximity duration, approach latency, and duration of stimulus interaction. Additionally, we applied a data-driven cluster analysis to investigate the occurrence of a wide range of other behaviours in response to the different stimuli.

**Results:**

Valence and activity levels did not affect cats’ responses to auditory stimuli. However, cats gazed longer and more frequently at negatively valenced visual stimuli depicting higher levels of activity compared to all other stimulus categories. Stimulus interaction was longer for active stimuli, independent of valence. However, cats displayed more fear-related behaviours in response to the negatively compared to positively valenced active stimuli, indicating that they interpreted these as being fundamentally different from each other.

**Conclusion:**

Our findings suggest that cat emotion perception in the visual domain is modulated by both valence and activity, highlighting the need to incorporate arousal in further emotion research.

## Introduction

Attributions of emotion play a critical role in survival. Emotions can be outwardly expressed through facial and bodily displays or vocalizations, offering valuable cues about the environment and social interactions (Feretti & Papaleo, 2019). These expressions can signal potential dangers in the environment or reveal significant information about a group member, prompting appropriate responses. It is widely assumed that sociality is a key prerequisite for perceiving emotions in conspecifics (Feretti & Papaleo, 2019, but see Kim & Kret, 2022). Historically, as descendants from the solitary-living African wildcat (*Felis silvestris lybica*), domestic cats (*Felis silvestris catus*) were thought of as asocial animals. Perhaps because of this assumption, felids are not often studied with regard to their socio-cognitive abilities (Benson-Amram, Griebling & Sluka, 2023). In particular, studies of their perception of conspecific emotions remains limited (Benson-Amram, Griebling & Sluka, 2023; Lea & Osthaus, 2018). However, the ability to discriminate and appropriately respond to cues of emotion in conspecifics is considered evolutionarily adaptive across species with a range of social structures, facilitating the detection and avoidance of threats but also coordination, care, and cooperation (Feretti & Papaleo, 2019; Darwin, 1872; Shariff & Tracy, 2011). In the current study, we provide a comprehensive exploration of intraspecific emotion perception across different modalities in domestic cats, extending what is known of their socio-cognitive abilities.

Emerging evidence suggests that the social lives of domestic cats may be more complex than previously believed (Crowell-Davis, 2007; Ley, 2016; Bradshaw, 2015; Brown & Bradshaw, 2014; Deputte et al., 2021) making them appropriate candidates for a study of emotion perception. Domestication facilitated intraspecific sociality in cats, which is reflected in their greater degree of social tolerance and higher frequencies of affiliative interactions compared to wildcats (Scott & Florkiewicz, 2023; Cameron-Beaumont, 1997). Similarly, free-living “feral” cats also form matrilineal social groups and display a flexible sociality (Crowell-Davis, 2007; Ley, 2016). Several studies have demonstrated that cats recognize family members and other familiar conspecifics across modalities (Bánszegi et al., 2017; Szenczi et al., 2016, 2022; Takagi et al., 2022) and that they display advanced social behaviours, such as communal maternal care and social learning (Crowell-Davis, 2007; Jaroš, 2017). Together, these findings underscore that cats are appropriate and timely candidates for research on emotion perception.

Discriminating between cues of emotion allows individuals to predict the behaviour of others and adjust their own responses accordingly. For such discrimination to occur, emotion states must be externally expressed and perceptible through at least one sensory modality—typically visual, auditory, or olfactory cues. Visually, cats can integrate facial movements (Scott & Florkiewicz, 2023; Bennett, Gourkow & Mills, 2017), body posture (Cameron-Beaumont, 1997), and position of tail and ears (Deputte et al., 2021; Cameron-Beaumont, 1997) into affective displays. In agonistic contexts, aggressive cats adopt postures that enhance their apparent size—arched back, piloerected fur, bristled tail, and erect, rotated ears—while maintaining direct eye contact. By contrast, defensive cats avert their gaze, crouch low with flattened ears, or expose their belly to de-escalate conflict (Crowell-Davis, 2007; Brown & Bradshaw, 2014; Leyhausen, 1979). Affiliative cues are also visually distinct: during friendly approaches, cats often hold their tail upright (“Tail Up”), a behaviour associated with reduced aggression and commonly observed when lower-ranking individuals approach higher-ranking ones (Cameron-Beaumont, 1997; Cafazzo & Natoli, 2009). Experimental evidence shows that cats approach silhouettes displaying this posture more quickly compared to those that do not, highlighting its function in affiliative interactions (Cameron-Beaumont, 1997). Tail Up appears to occur more often in cat-human interactions than in cat-cat interactions (Deputte et al., 2021), suggesting that social context may influence the occurrence of this behaviour.

In addition to visual cues, domestic cats use a wide vocal repertoire that further enables expression of emotion states. Vocalizations are often integrated with visual cues in overt displays of emotion (Schötz, 2019; Tavernier et al., 2020). Variations in vocal prosody can serve as an indicator of cats’ emotion states, including cues of both valence and arousal (Schötz, van de Weijer & Eklund, 2024). For example, aggressive cats may emit prolonged yowls, whereas defensive individuals may hiss when approached (Brown & Bradshaw, 2014; Tavernier et al., 2020). Although vocalizations are thought to be most common in agonistic encounters and mother-offspring interactions (Brown & Bradshaw, 2014), affiliative interactions, such as allorubbing, are often accompanied by purring. Additionally, trills may occur during friendly approaches towards conspecifics and humans (Crowell-Davis, 2007), although the full function of this vocalization in cat-cat communication remains unclear (Tavernier et al., 2020). Some vocalizations, like the meow, appear to be directed almost exclusively at humans (Moelk, 1944; Yeon et al., 2011), implying that the use of certain vocalizations strongly depends on the recipient.

Several studies have examined the production of emotion cues during social interactions; however, empirical research on how cats perceive these cues in conspecifics remains scarce. To date, most work has focused on cats’ responses to cues of human emotions (*e.g*., Humphrey et al., 2020; Galvan & Vonk, 2016; D’Ingeo et al., 2023). Although cats have even been reported to discriminate differently valenced human emotional odours (D’Ingeo et al., 2023), olfactory cues in intraspecific emotion communication have received little empirical attention–despite the species’ reliance on chemical signals for communication (Brown & Bradshaw, 2014). Only one study has directly investigated both intraspecific and interspecific emotion recognition in domestic cats (Quaranta et al., 2020). Here, cats showed a visual preference for congruent over incongruent audiovisual emotion stimuli and looked longer at a hissing cat than a purring one. However, the authors state that this effect may have been driven primarily by the salience of the “hiss” facial display. Whereas cat interactions normally include cues from more than one modality and multimodal stimuli remain essential to consider in emotion research, investigating cats’ reactions to unimodal stimuli can help to separately assess the impacts of facial movements and vocalizations on cats’ responses. Given that Quaranta et al. (2020) relied on a single set of stimuli, limiting generalizability, the question of how cats represent cues of intra- and inter-specific emotion also warrants further investigation.

The present study investigates how cats might discriminate unimodal cues of emotion by systematically observing their responses when presented with auditory or visual representations of conspecifics’ emotions that varied in both valence and arousal (Mendl, Burman & Paul, 2010; Russell, 2003). Whereas stimulus valence may determine a receiver’s behavioural response (Paul et al., 2020), the level of arousal conveyed by the stimulus may regulate the latency or vigour with which these actions are performed (Mendl & Paul, 2020). Involuntary physiological responses of the recipient, such as heart rate, skin conductance, and pupil dilation can be indicative of the degree of physiological activation or arousal that an individual experiences when confronted with a specific stimulus (Bradley et al., 2008; Kret et al., 2013; van Rooijen, Ploeger & Kret, 2017). Additionally, attentional biases—how quickly and persistently an animal orients toward emotionally salient stimuli—may reveal the subjective relevance of those cues to the receiver (Crump, Arnott & Bethell, 2018; Pool et al., 2016). We expected that if cats interpret emotion cues as meaningful, they should respond in ways that are adaptive from an evolutionary perspective. Specifically, negatively valenced or threatening stimuli—such as aggressive vocalizations or postures—may signal potential danger and thus, require heightened vigilance or avoidance. In contrast, positively valenced stimuli may indicate affiliative intent of a conspecific, making them more likely to be approached (Cameron-Beaumont, 1997; Paul et al., 2020). The present study initially aimed to investigate the following questions:

- Do cats display an attentional bias when confronted with differently valenced visual and/or auditory cues of conspecific emotion?
- Do cats display variance in approach behaviour and proximity towards sources of differently valenced visual and/or auditory cues of conspecific emotion?
- Do cats display variance in the time they spend interacting with sources of differently valenced visual and/or auditory cues of conspecific emotions?

We hypothesized that cats would gaze longer toward negatively compared to positively valenced stimuli, reflecting increased attention to a potential threat, and spend more time in proximity to positively valenced stimuli, reflecting approach behaviour. We also expected cats to be more likely to interact with positively valenced stimuli and avoid interactions with sources of negatively valenced ones. Although this was not included in our preregistered hypotheses, we investigated if we could identify clusters of behaviours and facial movements (coded with the *Facial Action Coding System* “CatFACS”; Caeiro, Burrows & Waller, 2017) that occur together by using a data-driven approach. We expected that the frequency with which cats display differently clustered behaviours would vary across stimuli of different categories. Furthermore, we explored how stimulus activity levels affected cats’ behavioural responses, as more active-appearing stimuli may be perceived as more arousing and thus elicit stronger reactions. This study offers new insights on how cats respond to the emotion expressions of conspecifics, contributing to a deeper and more nuanced understanding of feline social cognition and communication.

## Materials and Methods

### Subjects

This study included 24 healthy, mixed breed cats (12 male, 12 female) from 13 different households, with an average age of 6.21 years (SD = 3.73). Test subjects were recruited using snowball sampling and included cats of different ages, breeds, and backgrounds. None of the cats interacted with the experimenter on a regular basis. To participate, cats had to be neutered and at least 1 year of age. All cats were housed socially (Mean = 2.89 ± 1.37 cats per household), except for four individuals that lived in single-cat households at the time of testing.

### Stimuli

We tested the behavioural reaction of all cats towards both unimodal visual and auditory stimuli. For both modalities, we initially aimed to select four stimuli based on valence, capturing two different aspects of both positive and negative valence for generalizability. We therefore selected four visual displays (“Approach” (with Tail Up), “Roll,” “Back arch,” and “Crouch” (Table S1)) and four vocalizations (“Trill,” “Purr,” “Yowl,” and “Hiss” (Table S2)). We assessed stimulus valence based on the social context, either affiliative or agonistic, in which the depicted behaviours are normally displayed (Crowell-Davis, 2007; Brown & Bradshaw, 2014; Cameron-Beaumont, 1997; Schötz, 2019; Tavernier et al., 2020; Stanton, Sullivan & Fazio, 2015; Weiss, Mohan-Gibbons & Zawistowski, 2015). However, we realized, although not selected as such, that stimuli that vary in their behavioural context may convey different levels of arousal, which possibly affects the receiver’s reaction to the stimuli (Mendl & Paul, 2020). We therefore aimed to additionally categorize the stimuli on the levels of arousal they conveyed, incorporating a more dimensional approach (Mendl, Burman & Paul, 2010; Russell, 2003).

To validate the stimuli, we asked six animal behaviour experts to provide ratings of valence and arousal (rated as ‘intensity’) on a 7-point Likert scale. All but one of the experts had extensive experience with cats specifically; one veterinarian, three doctoral researchers, a postdoctoral researcher, and a professor, all with at least 2 years’ experience in this field. On a scale from 1 (very negative) to 7 (very positive), “Approach” (M = 6.11, SD ± 0.76) and “Roll” (M = 6.17, SD ± 0.79) visual stimuli were considered significantly more positively valenced than “Crouch” (M = 2.33, SD ± 1.08) and “Back arch” (M = 1.78, SD ± 0.55) (*ps* < 0.001). In addition, valence ratings did not differ between “Approach” and “Roll” (*p* = 0.91), or between “Crouch” and “Back arch” (*p* = 0.89). Auditory stimuli “Trill” (M = 5.78, SD ± 0.81) and “Purr” (M = 6.22, SD ± 1.00) were considered significantly more positively valenced than “Hiss” (M = 1.78, SD ± 0.81) and “Yowl” (M = 2.22, SD ± 1.31) (*ps* < 0.001). Valence ratings did not differ between “Trill” and “Purr” (*p* = 0.77), or between “Hiss” and “Yowl” (*p* = 0.54). Initially, we asked the same experts to rate arousal levels of the visual and auditory stimuli (Table S3). However, the experts’ ratings of the arousal levels of all stimuli varied significantly (Table S3).

As we could not reliably quantify arousal levels expressed by the cats based on ratings of the experts, we alternatively sought to make a behaviour-based distinction by categorizing stimuli according to their level of activity as a proxy for arousal (Russell, 2003; Dawson et al., 2019). In the present study, stimuli are considered *active* if these display, or are associated with, locomotion, explorative behaviour, dominance, and/or aggressive behaviour (“Approach”, “Trill”, “Back arch”, “Yowl”). *Passive* stimuli include stimuli that display, or are associated with, resting, avoidance, submission, and/or defensive behaviour (“Roll”, “Purr”, “Crouch”, “Hiss”). While we acknowledge that this active/passive distinction does not map perfectly on to a representation of arousal, it provides a functional way to describe differences in observable behavioural intensity. Moreover, we acknowledge that some of the selected behaviours may occur in different situations (*e.g*., cats may also purr when in pain (Tavernier et al., 2020)). Nevertheless, we believe that the main context for all selected behaviours has been described clearly enough in existing literature to categorize our stimuli accordingly (Crowell-Davis, 2007; Brown & Bradshaw, 2014; Cameron-Beaumont, 1997; Schötz, 2019; Tavernier et al., 2020; Stanton, Sullivan & Fazio, 2015; Weiss, Mohan-Gibbons & Zawistowski, 2015). Our dimensional approach of using valence and activity to categorize our stimuli resulted in four different stimuli categories per modality. Both visual and auditory stimuli were derived from freely available photographs on the Internet, good quality videos on YouTube, or collected from caregivers that responded to an online questionnaire and voluntarily videotaped social interactions of their cats.

For each behavioural category within both modalities, three exemplars representing that category were included. Visual stimuli encompassed life-sized cut-out photographs of cats that displayed their entire body posture. Cats have been reported to initially react to cat-shaped silhouettes as they would to a real unknown conspecific; *e.g*., by slowly approaching, sniffing, investigating, or vocalizing. Previous studies report cats sniffing the silhouettes from front to rear, before losing interest (Cameron-Beaumont, 1997). To make the stimuli in the present study even more realistic, we used full-colour images rather than silhouettes. These photographs were printed on cardboard and mounted on a wooden standard. Individual stimulus cats, which varied in coat colours and patterns, were represented only once in the stimulus set. Because of variation in body posture between stimuli categories, we used a distance of 3.5 cm between the eyes (measured in a live cat) as a benchmark, ensuring that all stimuli were correctly and equally sized. Auditory stimuli consisted of a single sound, which was played at the start of the trial. All vocalizations were made by different cats, varying in sex and age. Audio files were edited in Audacity (version 3.5.1.) to remove background noise and homogenize volume across categories. In actual social interactions, duration of vocalizations varies strongly between categories. To keep stimuli as naturalistic as possible, we did not standardize vocalization duration. As a result, the duration of some auditory stimuli (“Trill” and “Hiss”) was shorter than others (“Purr” and “Yowl”).

### Testing area

**Figure 1:**
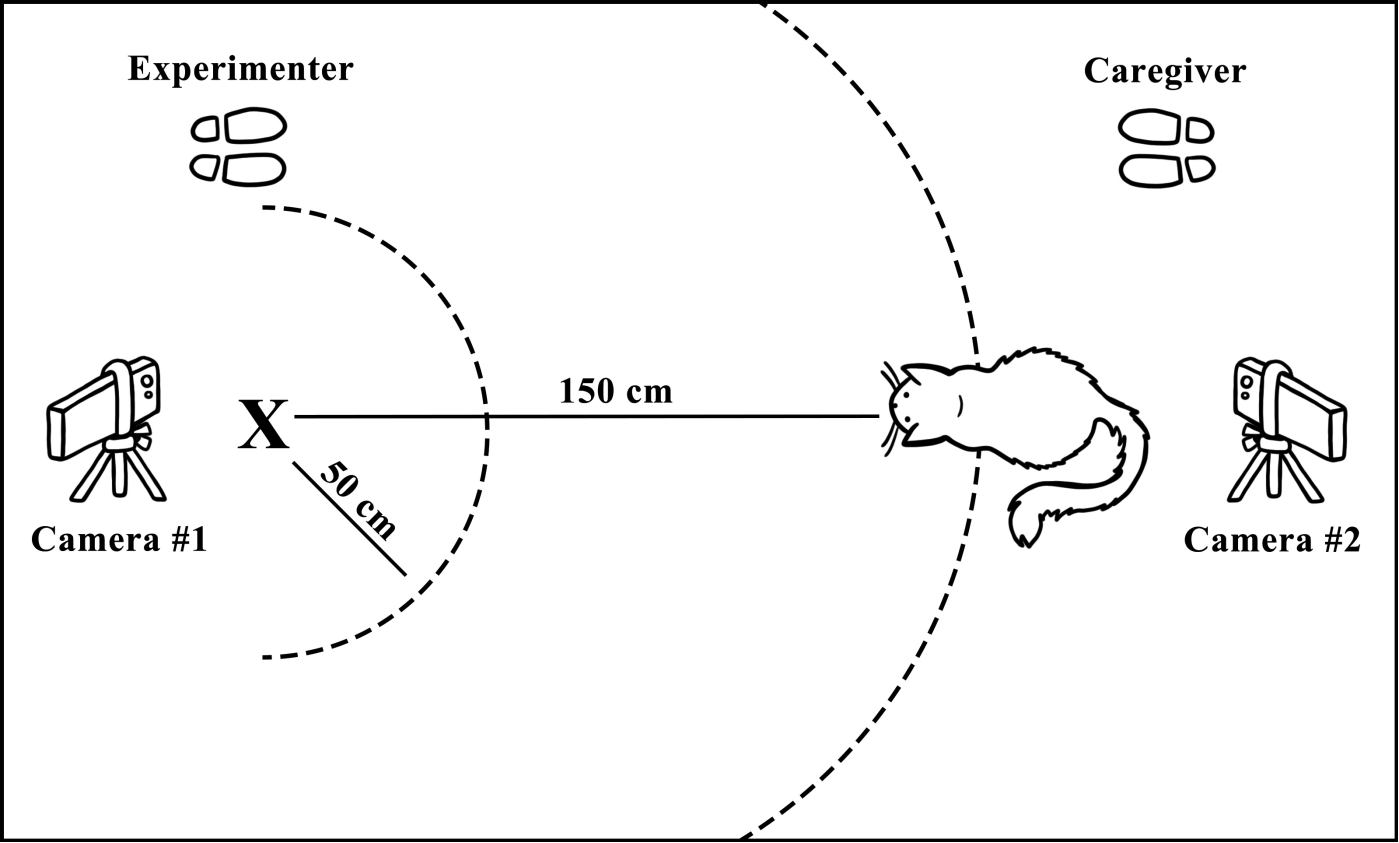
A top view of the global set-up of the testing area. The black “X” marks the location of the stimulus. Camera #3 is not depicted in this overview and is positioned further away from the testing area, filming the set-up from the side. Illustrated by Kiki Spoelstra.

Cats were tested in their own home in a familiar room that provided enough space to conduct the trials. Besides the test subject, their caregiver, and the experimenter, no other animals or humans were present in the room. We videotaped all trials from three angles: behind the stimulus, behind the cat’s starting point, and from the side. To determine proximity, markers were taped in a semicircle on the floor at a distance of 50 and 150 cm (Fig. 1). During the trials, the cats were always allowed to leave the testing area if they wanted to.

### Procedure

Test subjects participated in eight trials, four visual and four auditory, with each trial involving one randomly selected exemplar from each of the behavioural categories. Stimuli were presented in counterbalanced order across cats, with each cat receiving a different combination of stimuli within a session (Table S4). Each cat participated in at least two test sessions divided over different days. Typically, a test session included two visual trials and two auditory trials, resulting in a maximum of four trials per test session. Sessions always started with two trials of the same condition, either visual or auditory. On the following test day, a session started with a trial of the other condition. Although most cats were tested on two days, there were three cats that participated on three different days. This was due to cats leaving the house before the end of the session and a mistake in the test set-up.

Before the first session started, the caregiver was instructed and signed a consent form. In the meantime, we gave the cat at least 20 min to become habituated to the experimenter (KS). If a cat appeared relaxed (*e.g*., lying down in proximity) or had interacted in a positive manner with the experimenter, she prepared the trial in the assigned room. If this was not the case, *e.g*., individuals displaying signs of excessive stress (Kessler & Turner, 1997) or repeatedly fleeing from the experimenter upon her arrival, cats were excluded from participation. Next, the cat was allowed to explore the area, to reduce the likelihood that unknown objects and smells would affect their behaviour. When the cat finished inspecting the area or showed no reaction to the presence of the testing equipment, the first trial was conducted. The cat’s caregiver positioned the cat at the starting mark, 150 cm from the stimulus. They were instructed to hold the cat gently in the position and release them when the stimulus was presented. In the visual trials, the experimenter held a blank sheet of article in front of the stimulus to prevent the cat from seeing it before the trial started. Auditory stimuli were played from an external Bluetooth speaker (JBL Go), at a volume that represented that of a live vocalization. When the cat gazed in the general direction of the stimulus source, the experimenter lifted the sheet or started the playback, marking the start of the 2-min testing period. During the trial, the experimenter stood still to the side of the stimulus, just outside of the 50 cm range. The experimenter did not interact with the cat in any way. The caregiver was instructed to take a step back and to not interact with their cat (*e.g*., eye contact, talking, gesturing) during the trial. After 2 min, the experimenter ended the trial by removing the photograph or speaker. The caregiver was then allowed to interact with their cat while the experimenter prepared the next trial. A period of at least 2 min was implemented between two consecutive trials.

### Video analysis

We coded all videos of the trials in BORIS (version 8.24.1) (Friard & Gamba, 2016) according to a preestablished ethogram (Table S5), which included a wide variety of behaviours, including CatFACS action units (Caeiro, Burrows & Waller, 2017). FACS units were coded only when the cat was gazing at the stimulus until 1 s after the cat withdrew their gaze from the stimulus. We continuously coded gaze duration, close-contact interactions (*e.g*., sniffing, pawing) with the stimulus, proximity within 150 and 50 cm, approach latency and time spent out of sight. Gaze frequency and frequencies of visits between 50–150 cm and within 50 cm of the stimulus were collected as count data (Table 1). Lastly, we coded 1-0 occurrence of all other behaviours in the ethogram during the entire trial.

**Table 1:** All dependent variables included in statistical analysis, stimulus refers to either the photograph (visual condition) or the speaker that plays the vocalization (auditory condition).

Dependent variables	Description
*Continuous*	
Gaze duration (s)	Cat gazes in the direction of the stimulus, indicating attention.
Stimulus interaction duration (s)	Cat engages in a close-contact interaction with the stimulus, includes sniffing, pawing, rubbing, and cuffing.
Proximity duration (s)	Cat is located within 50–150 cm of the stimulus. Time is measured from the first moment the cat steps within the 150 cm range until all paws are out of the designated area.
Close proximity duration (s)	Cat is located within 50 cm of the stimulus. Time is measured from the first moment the cat steps within the 50 cm range until all paws are out of the designated area.
Approach latency (s)	The time that a cat takes to reach the stimulus to within 50 cm for the first time.
Out of sight duration (s)	The cat is not visible on any of the three video recordings.
*Count*	
Gaze frequency	The number of times a cat gazed in the direction of the stimulus during the entire test session.
Frequency proximity	The number of times a cat came within 50–150 cm of the stimulus during the entire test session
Frequency close proximity	The number of times a cat came within 50 cm of the stimulus during the entire test session.
Occurrence clustered behaviours	The number of behaviours from a specific clusters that occurred during the entire test session.

### Ethical statement

The set-up of the present study was approved by Leiden University’s Agency of Animal Welfare (Reference 23005) and the Psychology Ethics Committee (Reference 2024-01-24-M.E.Kret-V2-5137). Cats voluntarily participated in the study and were never restrained or forced to interact with the stimuli. We did not witness signs of excessive stress in the test subjects during or after testing (Kessler & Turner, 1997). All caregivers gave informed consent for their cats to participate.

### Inter-rater reliability

The videos of all trials (*n* = 192) were coded by the first author. A second coder (JY) who was naïve to the hypotheses coded 20% (*n* = 38) of the videos. We calculated agreement between the first and second coder for all behaviours and FACS action units; both coders were certified in CatFACS coding. For the continuous data, we calculated intra-class correlation coefficient (ICC) scores to test for inter-rater reliability (R-package *irr* (Koo & Li, 2016)). ICC scores ranged from 0.83 to 1.00 (Table S6A), which is considered good to excellent agreement (Koo & Li, 2016; Bateson & Martin, 2021). We calculated weighted Kappa coefficients (κ_w_) to estimate inter-rater agreement for gaze frequency and frequency of visits within 150 and 50 cm (Bateson & Martin, 2021; Harvey, 2021). Scores ranged from 0.73 to 0.93 (Table S6A), indicating good to excellent agreement (Fleiss, Levin & Paik, 2003).

Prevalence of individual behaviours varied greatly. For both modalities, only behaviours that occurred in at least 10% of the trials (Table S7 for selection) were analysed. Three behaviours (“Freeze,” “Piloerection,” and “Vocal (other)”) occurred in fewer than 10% of the auditory trials and were therefore included in the data analysis of only the visual condition. For the remaining behaviours, we employed two different methods for calculating inter-rater reliability. For behaviours that occurred in 10–90% of the randomly selected videos, we used the commonly applied Cohen’s Kappa (κ) statistic (Cohen, 1960). As this statistic is not always accurate when prevalence of a behaviour is either extremely low or high, we calculated prevalence-adjusted bias-adjusted Kappa (PABAK) for behaviours that occurred in >90% of the videos (Byrt, Bishop & Carlin, 1993). For our cluster analysis, we considered behaviours with a κ or PABAK of >0.40 (moderate agreement (Landis & Koch, 1977), Tables S6B, S6C). In some fields of research (*e.g*., healthcare studies) values below 0.60 are considered inadequate (McHugh, 2012). However, as the present study explores how clusters of behaviours differ between stimulus types and does not focus on individual behaviours, here a lower Kappa statistic may be acceptable.

### Data analysis

All statistical analyses were conducted in RStudio (version 2024.09.1), using R packages *lme4* (Bates et al., 2015), *ggplot2* (Wickham, 2016), *tidyverse* (Wickham et al., 2019) and multiple helper functions. We used mixed models for analysing the effect of stimulus valence, activity levels, and their interaction on the dependent variables (Table 1). For analysing the continuous variables, we applied a linear mixed model (LMM) and for the count data, a generalized linear mixed model (GLMM) with a log-link Poisson distribution. Specifically, we used the following model:

$\rm y = x \sim Valence * Activity + (1|Subject/Household).$

To account for the residuals not being normally distributed, we applied a log (x + 1) transformation before running the model. Resulting estimates and 95% confidence intervals are therefore depicted as odds ratios. If data were still zero-inflated after transformation, we applied a two-step *hurdle model* to perform the data analysis (Feng, 2021). This approach allowed us to first assess whether occurrence of a certain behaviour was affected by any of the fixed effects in our model. Next, we could assess whether these fixed factors affected duration or frequency of a behaviour, *if it occurred*.

To avoid testing all behavioural variables in separate models, increasing the chance of finding false positive effects, we grouped the behaviours that occurred together in a cluster analysis (Negri, 2022). This data-driven approach provided us with two dendrograms, one for each condition, that identified behaviours that occurred together more often (Bennett, Gourkow & Mills, 2017). For each cat, we used 1-0 sampling to identify which clustered behaviours were present or absent during each individual 2-min trial. The effects of stimulus valence, activity, and their interaction on the total occurrence of clustered behaviours were analysed in a similar GLMM as described above, using a Poisson distribution using a log-link function.

## Results

### Visual stimuli

#### Attention

**Figure 2:**
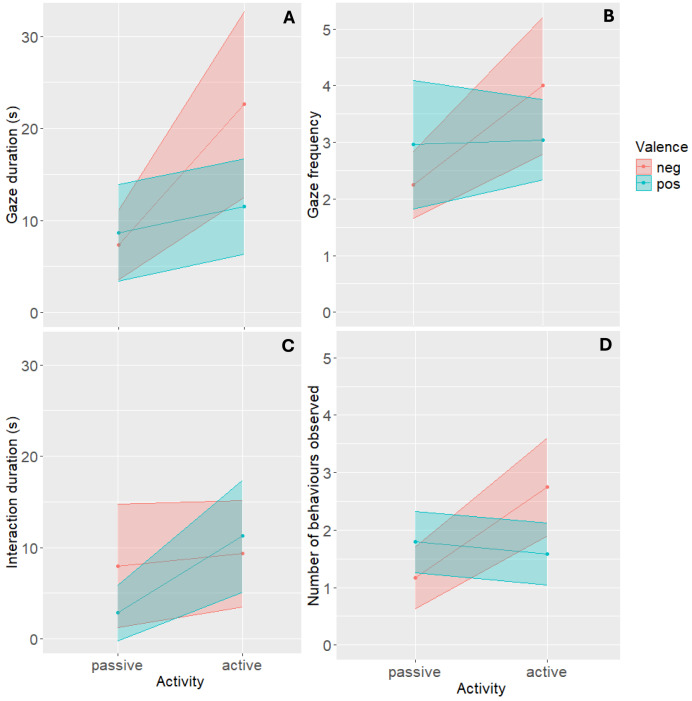
Interaction plots visualizing how the interaction between stimulus valence and activity affected cat behavioural responses in the visual condition. (A) Gaze duration. (B) Gaze frequency. (C) Interaction duration. (D) Occurrence of cluster 2 behaviours. The dots reflect the mean per group, the width of the ribbon reflects the 95% confidence interval.

Gaze duration towards the stimulus was significantly affected by an interaction effect between stimulus valence and activity (ß = 1.72, *t* = 2.22, *p* = 0.031, 95% CI [1.07–2.76]) (Fig. 2A). When presented with active stimuli, cats gazed longer at negatively valenced stimuli compared to positively valenced stimuli (ß = 1.90, *t* = 3.71, *p* < 0.001, 95% CI [1.36–2.65]). We found no difference in gaze duration between differently valenced passive stimuli. Pairwise comparisons revealed that cats gazed longer at the negatively valenced, active stimulus “Back arch” (Mean = 22.61, 95% CI [12.52–32.71]) compared to “Approach” (Mean = 11.53, ß = 0.53, *t* = −3.71, *p* = 0.0023, 95% CI [6.35–16.72]), “Crouch” (Mean = 7.33, ß = 0.38, *t* = −5.60, *p* < 0.001, 95% CI [3.55–11.10]), and “Roll” (Mean = 8.68, ß = 0.34, *t* = −6.17, *p* < 0.001, 95% CI [3.45–13.90]).

Gaze frequency was also affected by the interaction between stimulus valence and activity (ß = 1.73, *z* = 2.30, *p* = 0.022, 95% CI [1.09–2.76]) (Fig. 2B). Pairwise comparisons established that mean gaze frequency differed only between the two negatively valenced stimuli and was higher for “Back arch” stimuli (Mean = 4.00, 95% CI [2.79–5.21]) compared to “Crouch” stimuli (Mean = 2.25, ß = 0.56, *z* = −3.38, *p* = 0.0040, 95% CI [1.66–2.84]).

#### Stimulus interaction

Whether interaction with the stimulus occurred, was not affected by stimulus valence or an interaction effect with stimulus activity. However, *if* cats interacted with the stimulus, we found the interaction between these two fixed effects to affect its duration (ß = 0.41, *z* = 2.09, *p* = 0.043, 95% CI [0.18–0.93]) (Fig. 2C). Although we found no significant differences in interaction duration between positively and negatively valenced stimuli, pairwise comparisons revealed longer interaction times for active stimuli “Back arch” (Mean = 9.34, ß = 2.60, *t* = −2.96, *p* = 0.025, 95% CI [3.51–15.17]) and “Approach” (Mean = 11.26, ß = 3.36, *t* = −3.63, *p* = 0.0045, 95% CI [5.10–17.42]) compared to the positively valenced “Roll” stimuli (Mean = 2.86, 95% CI [0–5.92]).

#### Proximity

The frequency with which cats visited the stimulus within 150 or 50 cm proximity was not significantly affected by stimulus valence or its interaction with stimulus activity. Furthermore, we found no effect on proximity duration, both within 150 and 50 cm, and approach latency. We found no such effect on the occurrence of cats being out of sight or its duration.

#### Other behavioural measures

**Figure 3:**
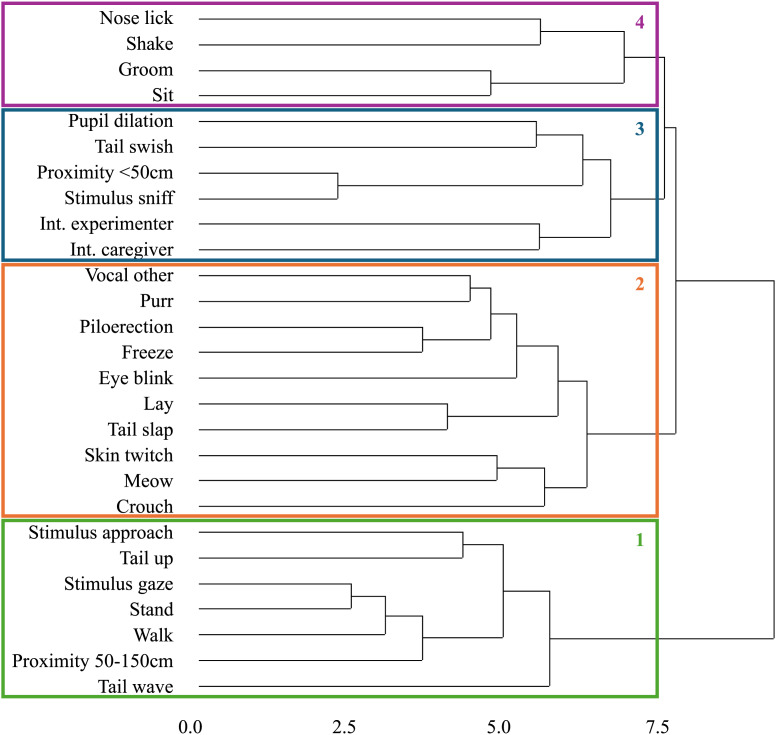
The dendrogram and resulting behavioural clustering in the visual condition. The x-axis represents the distance between the differently clustered behaviours. Numbers on the right vertical axis refer to the individual clusters.

To identify whether certain behaviours occurred together more often, we performed a cluster analysis based on 1-0 occurrence of the behaviours that were considered for data analysis. As there is no rule of thumb for determining the number of clusters from a dendrogram (Everitt et al., 2011), we adopted the conservative approach of applying one vertical cut through the middle of the longest branch, resulting in four different behavioural clusters (Fig. 3). From an ethological perspective, these predominantly align with cats displaying mild interest (Cluster 1), fear/arousal (Cluster 2), interest/arousal (Cluster 3), and frustration (Cluster 4) (Bennett, Gourkow & Mills, 2017; Stanton, Sullivan & Fazio, 2015; Weiss, Mohan-Gibbons & Zawistowski, 2015; Ellis, 2018; Nicholson & O’Carroll, 2021).

Occurrence of behaviours from Cluster 1, 3, and 4 was not affected by stimulus valence or its interaction with activity. However, we found a significant interaction effect of the two fixed effects on the total occurrence of behaviours from Cluster 2 (ß = 2.67, *z* = 3.10, *p* = 0.002, 95% CI [1.43–4.96], Fig. 2D). When presented with active stimuli, cats performed Cluster 2 behaviours more often for negatively valenced compared to positively valenced stimuli (ß = 1.74, *z* = 2.71, *p* = 0.007, 95% CI [1.17–2.59]). For passive stimuli, we found no such effect. Pairwise comparisons revealed that more Cluster 2 behaviours were observed when the cats were presented with a “Back arch” stimuli (Mean = 2.75, 95% CI [1.89–3.61]) compared to “Approach” (Mean = 1.58, ß = 1.74, *z* = 2.71, *p* = 0.034, 95% CI [1.04–2.12]) and “Crouch” (Mean = 1.17, ß = 2.36, *z* = 3.802, *p* < 0.001, 95% CI [0.63–1.70]).

### Auditory stimuli

#### Attention

We found no main effect of stimulus valence and no significant interaction effect of valence and activity on the cats’ gaze duration and gaze frequency towards the stimuli (Table S8).

#### Stimulus interaction

Whether stimulus interaction occurred was not significantly affected by stimulus valence or its interaction with stimulus activity. Furthermore, *if* interaction with the stimulus occurred, neither valence nor its interaction with stimulus activity affected duration (Table S8).

#### Proximity

Occurrence and duration of proximity within 150 and 50 cm were not affected by stimulus valence or its interaction with stimulus activity. Furthermore, we did not find any effect on approach latency and occurrence or duration of time that cats spent out of sight (Table S8).

#### Behavioural measures

Cluster analysis, based on 0-1 occurrence of behaviours in all auditory trials, identified only two different behavioural groupings (Fig. S9). Three behaviours, “Freeze,” “Vocal (other),” and “Piloerection”, occurred in less than 10% of the auditory trials and were not included. Possibly, this caused clusters to be less clear-cut than in the visual condition. The two behavioural groupings, especially Cluster 2, were large and not very informative from an ethological perspective. Therefore, we divided Cluster 2 into two separate clusters: 2a (frustration/arousal) and 2b (interest), based on the first subsequent branching in the dendrogram and existing literature (Bennett, Gourkow & Mills, 2017; Stanton, Sullivan & Fazio, 2015; Weiss, Mohan-Gibbons & Zawistowski, 2015; Ellis, 2018; Nicholson & O’Carroll, 2021). Nevertheless, we found no effect of stimulus valence or its interaction with stimulus activity on occurrence of behaviours from any cluster (Table S8).

## Discussion

The ability to perceive emotions is fundamental for all animals that engage in social interactions. Although domestic cats are often seen as independent or even aloof, such assumptions may underestimate their capacity to process socially relevant affective information. In the present study, we found partial support for the hypothesis that cats attend more closely to negatively compared to positively valenced emotion expressions of conspecifics, but only in the visual domain. Our results indicate that emotion stimuli characterized by both negative valence and elevated activity levels may be particularly salient, potentially signaling increased behavioural relevance in cat communication. Surprisingly, cats interacted longer with active stimuli regardless of valence, indicating that activity level alone may enhance social engagement. Whereas cats’ responses in the visual domain were modulated by both valence and activity level, the absence of such effects in the auditory domain suggests that these two modalities may play different roles in how cats perceive and respond to cues of emotion.

Both valence and arousal are considered important factors for animal affect (Mendl, Burman & Paul, 2010; Mendl & Paul, 2020). Although we could not include arousal conveyed by the stimuli in the present study, our results demonstrate that stimulus activity, having been suggested as a proxy for arousal (Dawson et al., 2019), also significantly modulates cat attentional biases towards expressions of emotion. Within an animal’s core affect space, perceived valence has been proposed to regulate selection of a behavioural action, whereas physical activation affects its strength (Mendl & Paul, 2020). This is in line with our observed interaction effect between stimulus valence and activity on cats’ gaze duration and their behavioural responses towards emotion visual stimuli. Within the visual domain, cats interacted with both positively and negatively valenced active stimuli. Distinct behavioural responses in response to differently valenced stimuli suggest that there may be various drivers for stimulus interaction. Vigilance and curiosity for novelty can increase attention, whereas lack of attention either reflects avoidance or reduced interest (Boissy et al., 2007). Both positively and negatively valenced high arousal situations may elicit increased alertness and activity (Mendl & Paul, 2020). However, whereas positively valenced affective states have gained more attention in recent years (Pool et al., 2016; Mellor, 2014), work on positively valenced high arousal states is practically non-existent (but see *positive contrast* (Mellor, 2014) and *positive affective engagement* (Öhman, Lundqvist & Esteves, 2001)). We would like to argue that the lack of corresponding literature hampers our full understanding of arousal in animal affect. Our results underline the need to further investigate both positively and negatively valenced arousal in future animal emotion research.

Cats use an extensive repertoire of visual displays to communicate affective state, including conspicuous facial movements and body postures (Deputte et al., 2021; Scott & Florkiewicz, 2023; Cameron-Beaumont, 1997; Bennett, Gourkow & Mills, 2017). It has been suggested that visual cues in cats primarily serve to regulate aggressive interactions (Brown & Bradshaw, 2014). We expected this to be reflected in cats’ behavioural responses towards differently valenced photographs of cats. Attentional biases towards emotion stimuli have been widely reported in humans (Yiend, 2010; Kano & Tomonaga, 2010) and nonhuman animals (Kappel et al., 2025), particularly for negatively valenced compared to neutral stimuli (*e.g*., chimpanzees (Kano & Tomonaga, 2010), rhesus macaques (Bethell et al., 2012; Lacreuse et al., 2013) and bonobos (van Berlo et al., 2024), but see Kret et al., 2016). We hypothesized that cats would show a similar bias towards negatively valenced visual stimuli, compared to positively valenced ones. However, our results demonstrate such a bias only for active, not passive, negatively valenced stimuli. A similar pattern was reported in a meta-analysis of human studies (Yuan et al., 2019), which found a stronger negativity bias for high-arousal compared to low-arousal stimuli—mirroring the interaction effect we observed for gaze duration. In general, it has been proposed that attentional biases towards emotion stimuli may depend more on their arousal-eliciting capacity than on valence (Russell, 2003; Pool et al., 2016; Anderson, 2005; Zsidó, 2024). For cats, encountering a conspecific that displays a “Back arch” posture may elicit heightened arousal and thus, increased attention. In contrast, a “Crouch” posture may be perceived as less immediately threatening and therefore less salient. Similarly, positively valenced stimuli–even when active–may not evoke the same level of arousal, as these are not likely to signal a threat.

Unexpectedly, cats interacted longer with active stimuli independent of their valence within the visual domain. This seems somewhat counterintuitive where negatively valenced stimuli are concerned, as these commonly elicit an avoidance response instead (*e.g*., Wathan et al., 2016). Still, it has been proposed that stimulus intensity and relevance are often correlated (Northoff et al., 2009). Whereas passive stimuli may be safe to ignore, both positively and negatively valenced active stimuli may be perceived as more intense and elicit a stronger need to react, either by approaching or avoiding. Additionally, inspection of a potentially dangerous or ambiguous cue can serve as a method to further assess the threat level of a stimulus (Mendl & Paul, 2020; Paul, Harding & Mendl, 2005; Fishman, 1999). Although domestic cats do not have a strict social hierarchy (Bradshaw, 2015), it is possible that active stimuli more closely approximated signals of dominance, rather than subordination. Such behaviours may play a role in determining the outcome of cat encounters; whereas subordinate cats are more likely to avoid aggressive interactions, more dominant individuals more often engage in a stare-down or approach while exhibiting a dominance display (Crowell-Davis, 2007). Duration of stimulus interaction may therefore not reliably indicate whether an individual correctly evaluates a conspecific’s emotional valence, but it could be reflective of the relevance of an expression within cat communication. Future work should evaluate how dominant and subordinate cats may differ in response to each other’s signals. An individual cat’s history and experience with other cats also likely plays a role in how that individual perceives and responds to conspecific signals. All but four of the cats in the current study resided with at least one other cat, but future studies should examine how cats’ rearing histories and social experiences influence their responses to species-typical emotion displays.

How cats perceive a specific expression of emotion may also be reflected in the occurrence of specific behavioural patterns. In a data-driven approach, we identified four clusters of behaviours that occurred together more often when cats were presented with visual stimuli. Occurrence of behaviours from one specific cluster was strongly affected by the interaction of stimulus valence and activity. This cluster predominantly included behaviours associated with fear and arousal, such as piloerection, vocalizing, freezing, tail slapping, skin twitching and crouching (Bennett, Gourkow & Mills, 2017; Stanton, Sullivan & Fazio, 2015; Weiss, Mohan-Gibbons & Zawistowski, 2015; Ellis, 2018; Nicholson & O’Carroll, 2021). Although we expected such behaviours to occur more often when cats were presented with both negatively valenced stimuli, this was true only for active stimuli. Whereas both positively and negatively valenced active stimuli were interacted with, a difference in general behavioural response suggests that cats still perceived these unimodal emotion stimuli as being fundamentally different.

Domestic cats have an extensive vocal repertoire, with adult vocalizations thought to serve mainly agonistic or sexual functions (Brown & Bradshaw, 2014; Tavernier et al., 2020). Mirroring our expectations for the visual stimuli, we predicted cats to be more attentive towards negatively valenced vocalizations compared to positively valenced vocalizations, regardless of activity levels. In addition, we predicted longer stimulus interaction and proximity durations for positively compared to negatively valenced sounds. Contrary to those expectations, stimulus valence and activity had no impact on gaze duration, approach behaviour, or proximity duration. Instead, cats displayed only brief attention to playback sounds—a pattern that may reflect the lack of complementary visual or olfactory context in our setup. This aligns with previous research, which reported cats responding faster to both visual and bimodal cues compared to isolated auditory ones (De Mouzon, Di-Stasi & Leboucher, 2023). Additionally, short call durations may have been insufficient to elicit sustained attention over the 2-min trial. Measures like gaze duration, stimulus interaction and proximity may therefore not be suited for assessing attention to auditory stimuli. Notably, we often observed cats pausing to scan the room or sniff the floor immediately after hearing the playback. These exploratory behaviours–though not included in our ethogram–may represent attempts to gather additional sensory information to shape an appropriate behavioural response. Incorporating such behaviours in future work may be helpful in assessing the relevance of auditory signals.

Within the wide variety of emotion expressions that domestic cats display across modalities, some cues may be more important than others (Mayes et al., 2015). In the present study, visual stimuli were much more successful in eliciting sustained attention compared to auditory stimuli. In previous literature, it has been proposed that vocalization may not be as important as visual displays in cat social interactions (Brown & Bradshaw, 2014; Cameron-Beaumont, 1997). Still, cat vocalizations are likely to convey information about emotion. Vocal parameters indicating arousal in mammals, including cats, have been extensively researched, although acoustic correlates of valence are not as well defined yet (Briefer, 2020, 2012 for review). Furthermore, cats have been reported to integrate acoustic and visual signals into a multimodal perception of human and conspecific emotion (Quaranta et al., 2020). To further investigate more subtle effects of valence and arousal in feline vocalization, pairing playbacks with congruent signals from other modalities may be necessary. Although not included in the present study, olfactory cues could be especially important and should be investigated in future studies on cat emotion perception (Brown & Bradshaw, 2014).

### Limitations

In the present study, among other behaviours, we aimed to investigate the FACS action units that are of interest in the cats’ reaction to different emotion stimuli. However, inter-rater reliability for most action units was low and only three (pupil dilation, nose lick, and eye blink) could be reliably assessed in our video footage. In particular, when a cat gazed at the stimulus from further away, coding smaller facial movements was challenging, if not impossible. Future studies that aim to incorporate CatFACS should ensure that all movements can be coded reliably. Another limitation of this study, is that animal behaviour experts strongly disagreed on arousal ratings of both visual and auditory stimuli. Previous work has demonstrated that humans performed just above chance when asked to correctly assess the valence of cat emotions based on unimodal visual stimuli (Dawson et al., 2019; Bouma et al., 2024). Conversely, it has been demonstrated that humans can identify high arousal vocalizations across species (Filippi et al., 2017), whereas vocal correlates of emotional valence remain unclear (Briefer, 2020, 2012). However, previous cat studies have mostly focused on the intensity of ‘meow’ vocalizations in different contexts (Schötz & van de Weijer, 2014; Nicastro & Owren, 2003) (but see (McComb et al., 2009) for ‘purr’). To our knowledge, it has not yet been investigated whether humans can reliably recognize arousal in other cat vocalizations. Research such as the present study may further elucidate the nature of different signals in cat communication, which will also help humans to interpret these more accurately. Further limitations of the current work involve the relatively small sample size and stimulus sets. Possibly, transforming the data due to normality concerns may have flattened the natural variability in the responses, reducing our ability to detect significant effects. Furthermore, we used snowball sampling, which could have led to a biased sample. It is possible that the cats selected for inclusion were more gregarious or calmer than cats that had to be excluded or whose caregivers did not permit their participation. Therefore, future work is necessary to determine the generalizability of our results.

## Conclusions

The results of the current study further our limited understanding of intraspecific emotion perception in domestic cats within the visual and auditory domains. We hypothesized that domestic cats would react differently to cues of conspecific emotions depending on the valence of such stimuli. However, cats’ behavioural responses towards visual stimuli were especially affected by the interaction between stimulus valence and activity, indicating that both factors contribute to informing an appropriate behavioural response. Reactions towards auditory stimuli were not as clear-cut, suggesting that additional information from other sensory domains may be required for correctly interpreting conspecific emotions from this modality. For future animal emotion studies, we strongly advise researchers to investigate the effect of both valence and arousal. We recognize that determining the level of arousal that is conveyed by specific stimuli can be challenging; therefore, we suggest that stimulus activity may stand in for at least one component of arousal. Therefore, future recommendations are to further investigate methods to include measures of arousal in animal emotion research and, specifically, to focus on the possible differences between positively and negatively valenced high arousal states.

## Supplemental Information

10.7717/peerj.21292/supp-1 Supplemental Information 1 The complete stimulus set used in the visual condition.

10.7717/peerj.21292/supp-2 Supplemental Information 2 Example spectrograms for the different types of auditory stimuli. The x-axis is indicative of the duration (s) of the stimulus and the y-axis represents frequency (Hz). Colour range depicts amplitude of the sound; here, lighter colours reflect higher amplitudes.

10.7717/peerj.21292/supp-3 Supplemental Information 3 Mean intensity ratings ± SD per stimulus type, rated by animal behaviour experts (N=6).

10.7717/peerj.21292/supp-4 Supplemental Information 4 Flowchart depicting the order of stimulus presentation over all trials.

10.7717/peerj.21292/supp-5 Supplemental Information 5 Ethogram describing the full set of behaviours that were observed during video analysis. Descriptions for the vocalizations are derived from Tavernier et al. (Tavernier et al., 2020). Descriptions for the general behaviours are adapted from Stanton et al. (Stanton, Sullivan & Fazio, 2015). If no body posture or tail position is coded, it can be assumed to be neutral. Descriptions of FACS behaviours were taken from the CatFACS manual (freely available on www.CatFACS.com).

10.7717/peerj.21292/supp-6 Supplemental Information 6 ICC and weighted kappa for all duration and count behaviours.

10.7717/peerj.21292/supp-7 Supplemental Information 7 Cohen’s Kappa and PABAK scores for all behaviours and FACS action units that were included in the final data analysis. Behaviours that are not listed either occurred in <10% of the observations or could not be reliably coded ( κ or PABAK <0.40). Behaviours in italics were only included in the trials in the visual condition.

10.7717/peerj.21292/supp-8 Supplemental Information 8 All behaviours and FACS units that were omitted because of low inter-rater reliability (κ <0.40) and their corresponding κ-statistics.

10.7717/peerj.21292/supp-9 Supplemental Information 9 Ethogram containing all behaviours that were considered for the cluster analysis. Descriptions of the general behaviours were adapted from Stanton et al. (Stanton, Sullivan & Fazio, 2015), descriptions of the vocalizations were adapted from Tavernier et al. (Tavernier et al., 2020), and descriptions of FACS behaviours were taken from the CatFACS manual (freely available on www.CatFACS.com).

10.7717/peerj.21292/supp-10 Supplemental Information 10 Full results table for both the visual and the auditory condition. ^a^ Results from step 1 of the hurdle model (GLMM binomial), ^b^ Results from step 2 of the hurdle model (GLMM with gamma distribution). •p<0.1, *p<0.05, **p<0.01, ***p<0.001.

10.7717/peerj.21292/supp-11 Supplemental Information 11 The dendrogram and resulting behavioural clustering from the trials in the auditory condition. The x-axis represents the distance between the differently clustered behaviours. Numbers on the right vertical axis refer to the individual clusters.

10.7717/peerj.21292/supp-12 Supplemental Information 12 All auditory stimuli used in the present study.

## Additional Information and Declarations

### Data Availability

The following information was supplied regarding data availability:

The data that support the findings of this study are available in DataverseNL at https://doi.org/10.34894/EYN5Y7.

- Spoelstra, Kiki, Jennifer Vonk, Evy van Berlo, José; Kok, and Mariska Kret. 2026. “Purr-Ceiving Feelings: Domestic Cats Respond to Intraspecific Cues of Emotion.” DataverseNL.
