# Supplementary material for: Purr-ceiving feelings: domestic cats respond to intraspecific cues of emotion
Source: PeerJ. 2026 May 25;14:e21292. doi: 10.7717/peerj.21292 (PMC13218337; doi:10.7717/peerj.21292)
Supplement: Supplemental Information 1 [file peerj-14-21292-s001.pdf]

	Positive			***Negative***		
Passive	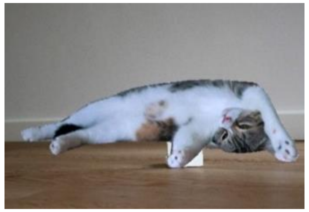	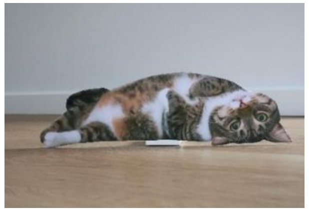	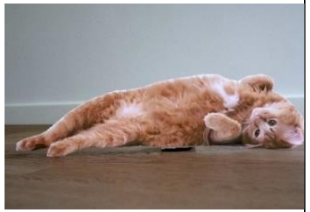	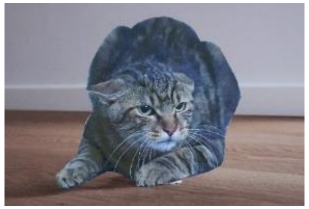	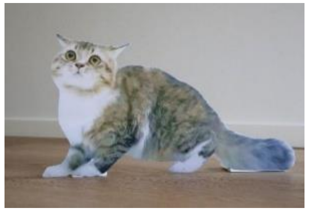	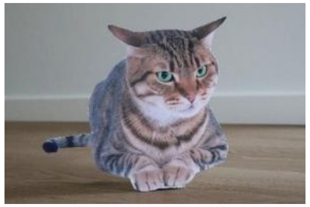
	• The cat’s belly is exposed<br>• The entire body of the cat is visible<br>• The entire body of the cat is resting on the floor			• The cat’s body is lowered and the legs are bent<br>• The cat’s ears are flattened or rotated<br>• The cat’s eyes are open, the pupils may be dilated		
Active	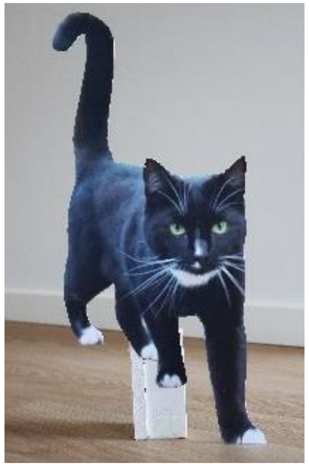	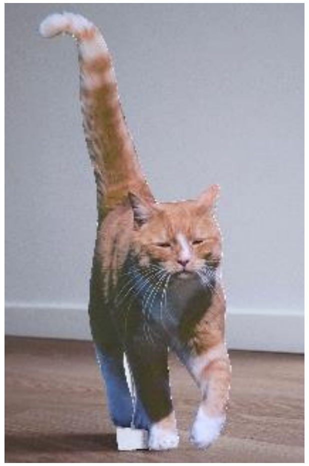	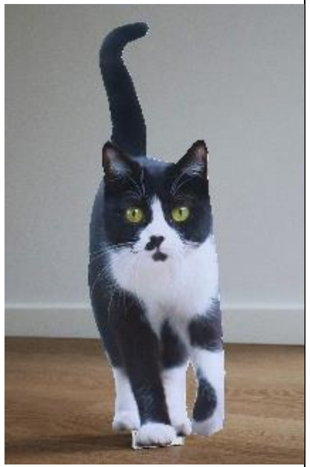	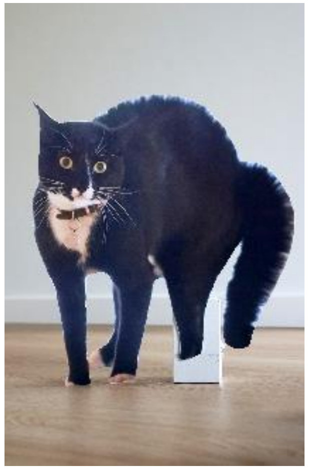	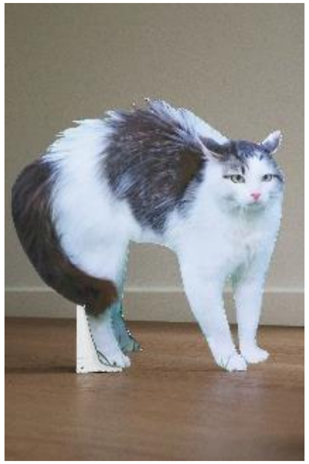	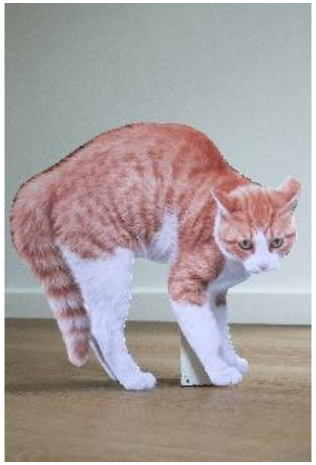
	• The cat’s tail is pointing up, the point is bent to the side<br>• The cat is walking towards the viewer<br>• The cat’s ears are clearly visible and not flattened			• The cat’s body is arched and orientated to the side<br>• The hairs on the cat’s back and/or tail are erected<br>• The cat’s ears are flattened or rotated<br>• The cat’s eyes are open, the pupils may be dilated		
