# Supplementary figures and images for: Purr-ceiving feelings: domestic cats respond to intraspecific cues of emotion
Source: PeerJ. 2026 May 25;14:e21292. doi: 10.7717/peerj.21292 (PMC13218337; doi:10.7717/peerj.21292)

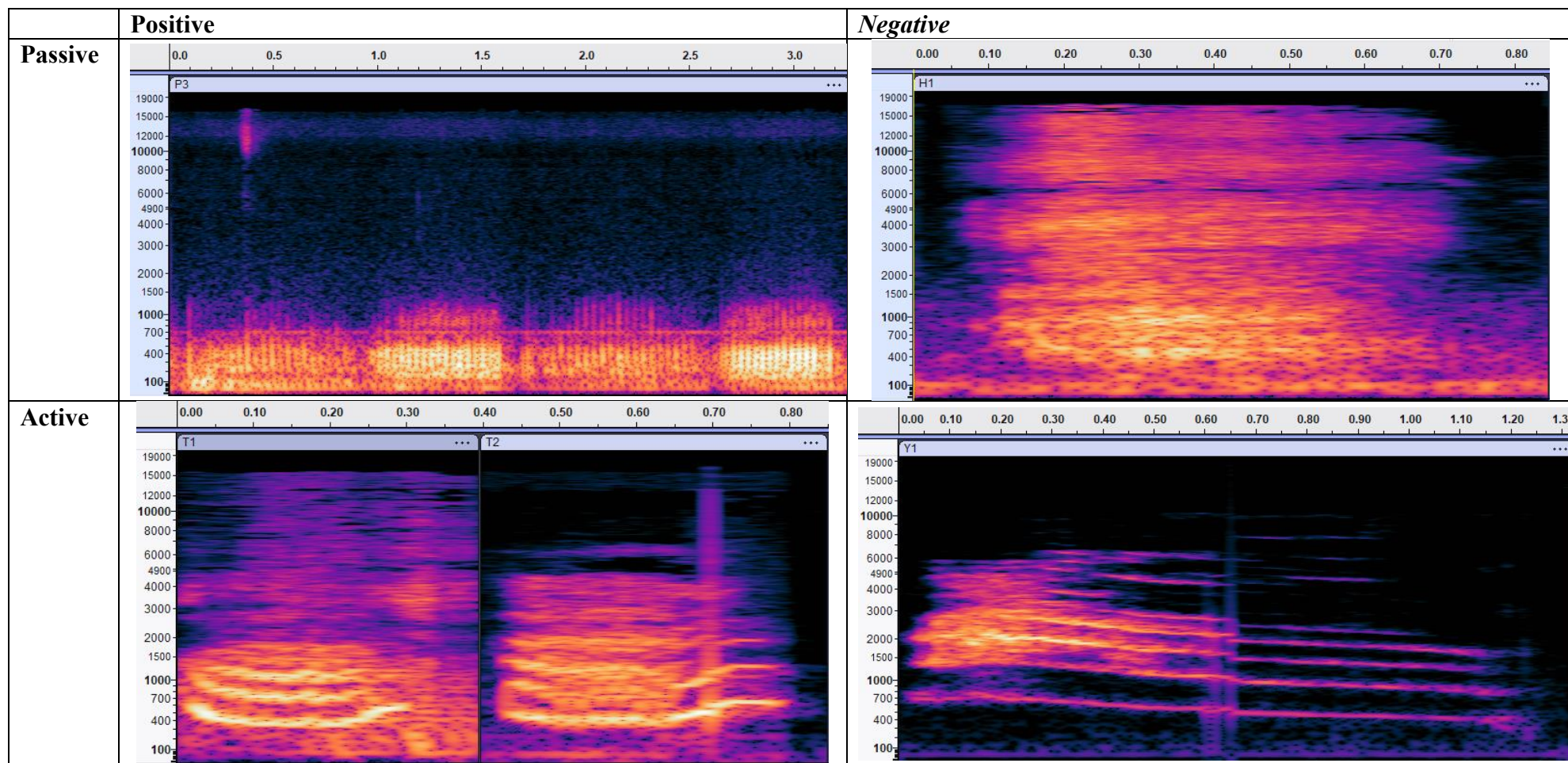

Supplement: Supplemental Information 2 — The x-axis is indicative of the duration (s) of the stimulus and the y-axis represents frequency (Hz). Colour range depicts amplitude of the sound; here, lighter colours reflect higher amplitudes. [file peerj-14-21292-s002.pdf]

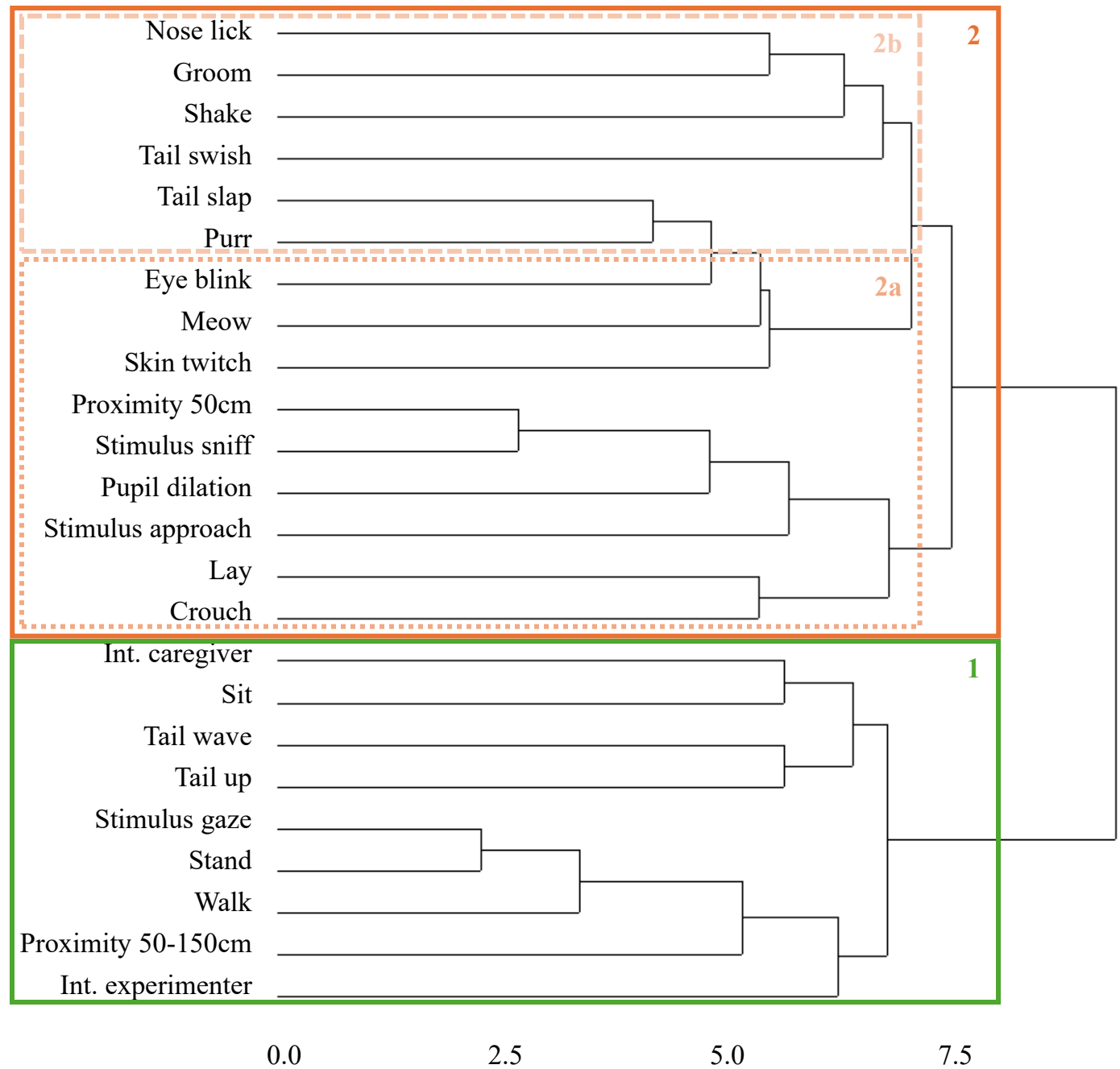

Supplement: Supplemental Information 11 — The x-axis represents the distance between the differently clustered behaviours. Numbers on the right vertical axis refer to the individual clusters. [file peerj-14-21292-s011.pdf]
