# Supplementary material for: Purr-ceiving feelings: domestic cats respond to intraspecific cues of emotion
Source: PeerJ. 2026 May 25;14:e21292. doi: 10.7717/peerj.21292 (PMC13218337; doi:10.7717/peerj.21292)
Supplement: Supplemental Information 3 [file peerj-14-21292-s003.pdf]

**Stimulus**	**Valence**	**Mean**	**SD**
Visual			
Roll	Positive	2.17	1.29
Approach	Positive	2.78	1.93
Crouch	Negative	5.00	1.46
Back arch	Negative	5.56	1.29
Auditory			
Purr	Positive	4.89	1.45
Trill	Positive	4.78	1.40
Hiss	Negative	4.89	1.60
Yowl	Negative	5.56	1.04
