# Supplementary material for: Purr-ceiving feelings: domestic cats respond to intraspecific cues of emotion
Source: PeerJ. 2026 May 25;14:e21292. doi: 10.7717/peerj.21292 (PMC13218337; doi:10.7717/peerj.21292)
Supplement: Supplemental Information 4 [file peerj-14-21292-s004.pdf]

Day 1				Day 2			
Trial 1	Trial 2	Trial 3	Trial 4	Trial 5	Trial 6	Trial 7	Trial 8
**VIS –** → N=6 cats	**VIS +** → / →	**AUD +** → N=3 cats	**AUD -** →	**AUD -** →	**AUD +** →	**VIS +** → N=6 cats	**VIS -**
		**AUD -** → N=3 cats	**AUD +** →	**AUD +** →	**AUD -** →		
**VIS +** → N=6 cats	**VIS -** → / →	**AUD +** → N=3 cats	**AUD -** →	**AUD -** →	**AUD +** →	**VIS -** → N=6 cats	**VIS +**
		**AUD -** → N=3 cats	**AUD +** →	**AUD +** →	**AUD -** →		
**AUD -** → N=6 cats	**AUD +** → / →	**VIS +** → N=3 cats	**VIS -** →	**VIS -** →	**VIS +** →	**AUD +** → N=6 cats	**AUD -**
		**VIS -** → N=3 cats	**VIS +** →	**VIS +** →	**VIS -** →		
**AUD +** → N=6 cats	**AUD -** → / →	**VIS +** → N=3 cats	**VIS -** →	**VIS -** →	**VIS +** →	**AUD -** → N=6 cats	**AUD +**
		**VIS -** → N=3 cats	**VIS +** →	**VIS +** →	**VIS -** →		
