# Supplementary material for: Purr-ceiving feelings: domestic cats respond to intraspecific cues of emotion
Source: PeerJ. 2026 May 25;14:e21292. doi: 10.7717/peerj.21292 (PMC13218337; doi:10.7717/peerj.21292)
Supplement: Supplemental Information 5 — Descriptions for the vocalizations are derived from Tavernier et al. (Tavernier et al., 2020). Descriptions for the general behaviours are adapted from Stanton et al. (Stanton, Sullivan & Fazio, 2015). If no body posture or tail position is coded, it can be assumed to be neutral. Descriptions of FACS behaviours were taken from the CatFACS manual (freely available on www.CatFACS.com). [file peerj-14-21292-s005.docx]

| **Behaviour** | **Description** |
| --- | --- |
| *Stimulus interaction* |  |
| Attack | Cat launches itself at stimulus and attempts to engage in physical combat. |
| Cuff | Cat hits stimulus with its forepaw(s), claws are extracted. |
| Bite/chew | Cat bites stimulus material or chews on it. |
| Flee | Cat runs away from stimulus. |
| Paw | Cat pats stimulus with its forepaw(s). Claws are usually retracted. |
| Play | Cat interacts with stimulus in “non-serious” manner, i.e. there is no intention to harm. |
| Retreat | Cat backs away from stimulus while watching it. |
| Rub | Cat rubs head or any part of body against stimulus. |
| Sniff | Cat sniffs stimulus by inhaling air through the nose from within 50 cm. |
| *Static* |  |
| Lay | Cat’s body is on the ground in a horizontal position on its side, back or belly. |
| Sit | Cat is in an upright position, with the hind legs flexed and resting on the ground, while front legs are extended and straight. |
| Stand | Cat is in an upright position and immobile, with all four paws on the ground and legs extended, supporting the body. |
| *Locomotion* |  |
| Walk | Cat locomotes forward at a slow gait. |
| Trot | Cat locomotes forward at a swift gait performed with alternating steps. Movement is faster than walking but slower than running. |
| Run | Cat locomotes forward in a rapid gait, which is faster than walking or trotting. |
| Slink | Cat walks with chest and abdomen close to the ground and legs bent. |
| *Body posture* |  |
| Back arch | Cat stands rigidly and arches back upwards. |
| Crouch | Cat is alert and positions the body close to the ground, whereby all four legs are bent and the belly is touching (or raised slightly off of) the ground. |
| Freeze | Cat suddenly becomes immobile, body is tense. |
| *Tail position* |  |
| Neutral | Cat holds tail downward but relaxed, may be slightly bent. |
| Quiver | Cat vibrates part of/entire tail while raising it up or half-up. |
| Slap | Cat quickly strikes its tail on the ground. |
| Swish | Cat violently swishes the tail. |
| Tucked | Cat tucks tail right under the body. |
| Twitch | Cat flicks the tail in either a side to side or up to down motion. |
| Up | Cat holds the tail in an upright position. The tip may be slightly curved. |
| Wave | Cat slowly and gently waves the tail from side to side. |
| *Vocalization* |  |
| Growl | Low-pitched, throaty noise made with a closed or slightly opened mouth. |
| Hiss | Low-intensity hissing sound produced by an expulsion of air from the opened mouth of the cat. |
| Meow | The distinctive “meow” call that is typical of cat, the mouth is opened and closed gradually. |
| Purr | Continuous murmuring sound, produced during respiration with a closed mouth. |
| Trill | Short, soft-voiced trilling or purring sound made with a closed mouth. |
| Yowl | Long, drawn-out vocalization that varies in pitch, intensity, duration and tonality. |
| Vocal (other) | Any vocalization that cannot be categorized as a growl, hiss, meow, purr, trill, or yowl. |
| *Other behaviours* |  |
| Body/head shake | Cat rotates its head, a body part or the entire body from side to side. |
| Flehmen | Cat makes a grimaced facial expression, where the mouth is open, upper lip is elevated and tongue may protrude out of the mouth. |
| Groom | Cat cleans itself by licking, biting, or chewing the fur on its body. May include the licking of a front paw and wiping it over one’s head. |
| Interaction owner | Cat seeks contact with owner by gazing, vocalizing while gazing/approaching or seeking physical contact. |
| Interaction exp. | Cat seeks contact with experimenter by gazing, vocalizing while gazing/approaching or seeking physical contact. |
| Object play | Cat interacts with inanimate object in a “non-serious” manner. |
| Piloerection | Cat raises the hairs on the nape of its neck, shoulder, back or tail, so that the fur is standing erect. |
| Roll | Cat rotates body from one side to another while lying on its back on the ground. Belly is exposed. |
| Scratch | Cat uses the claws of its hind feet to scratch its body. |
| Skin twitch | Cat quickly twitches its skin near the base of the tail in a small amplitude movement. |
| Urine spray | Cat releases a jet of urine backwards while standing with the tail raised vertically. |
| Yawn | Cat opens its mouth widely while inhaling, then closes the mouth while exhaling deeply. |
| Out of sight | Cat is not visible in any of the three videos. |
| **FACS action unit** | **Description** |
| Ears forward (EAD101) | The ears move cranially in a small amplitude movement. A small decrease in the space between ears and eyes may be noticeable. |
| Ears adducted (EAD102) | The ears are pulled towards the midline, decreasing the space between them. |
| Ears flat (EAD103) | The ears are half-rotated and pulled caudally and towards the occipital region, decreasing the ear opening. Ear cartilage is folded and ear opening is covered. |
| Ears rotated (EAD104) | The ears are rotated and the ear opening is turned laterally. The caudal surface of the ears rotates medially. Can be distinguished from EAD103 by the absence of cutaneous pouch folding. |
| Ears down (EAD105) | The ears are pulled ventrally and slide laterally in a small amplitude movement, increasing the distance between the ears. |
| Ears back (EAD106) | The ears swivel caudally in a small amplitude movement, appearing taller and enlarging the ear opening. |
| Ears constrict (EAD107) | The lateral and medial margins of the ear move towards each other, decreasing the opening of the ear. The ears appear slender, taller and pointier. |
| Eye closure (AU143) | The upper and lower eyelids move towards each other, eventually closing the eye. The eye remains closed for half a second or more. |
| Eye blink (AU145) | The upper and lower eyelids move towards each other, eventually closing the eye. The eye opens within half a second. |
| Half blink (AU47) | The upper and lower eyelids move towards each other, without closing the eye. |
| Upper lid raiser (AU5) | The upper eyelid is pulled dorsally, further exposing the eyeball. The pupil becomes completely visible, the iris become more exposed. |
| Lip wipe (AD37) | The tongue wipes the lips from the mouth midline to the mouth corner in a lateral movement. |
| Nose lick (AD137) | The tongue is moved beyond the lips and in a dorsal movement, wiping the nose. |
| Pupil dilation (AD68) | Pupil increases in size. |
| Pupil constriction (AD69) | Pupil decreases in size. |
| Whisker retraction (AU200) | The whiskers swivel caudally, may lie against the side of the face. |
| Whisker protraction (AU201) | The whiskers swivel cranially, may curl towards each other. |
