# Supplementary material for: Purr-ceiving feelings: domestic cats respond to intraspecific cues of emotion
Source: PeerJ. 2026 May 25;14:e21292. doi: 10.7717/peerj.21292 (PMC13218337; doi:10.7717/peerj.21292)
Supplement: Supplemental Information 6 [file peerj-14-21292-s006.pdf]

**Behaviour (duration)**	**ICC**
Gaze duration	0.95
Interaction duration	0.84
Proximity duration	0.83
Close proximity duration	1.00
Approach latency	1.00
Out of sight duration	0.90

**Behaviour (count)**	**K_w**
Frequency proximity	0.93
Frequency close proximity	0.92
Gaze frequency	0.73
