# Supplementary material for: Purr-ceiving feelings: domestic cats respond to intraspecific cues of emotion
Source: PeerJ. 2026 May 25;14:e21292. doi: 10.7717/peerj.21292 (PMC13218337; doi:10.7717/peerj.21292)
Supplement: Supplemental Information 7 — Behaviours that are not listed either occurred in <10% of the observations or could not be reliably coded ( κ or PABAK <0.40). Behaviours in italics were only included in the trials in the visual condition. [file peerj-14-21292-s007.pdf]

Behaviour/FACS	Cohen's κ
Meow	0.83
Tail up	0.62
Tail swish	0.42
Tail wave	0.50
Lay	0.89
Sit	0.88
Crouch	0.42
Shake	0.80
Groom	0.89
Interaction owner	0.79
Interaction exp.	0.56
Skin twitch	0.48
Stimulus approach	0.76
Stimulus sniff	1.00
AD137 (nose lick)	0.65
AD68 (pupil dilation)	0.53
AU145 (eye blink)	0.52

Behaviour	Byrt's PABAK
Purr	0.74
Vocal (other)	*0.89*
Tail slap	0.68
Freeze	*0.68*
Walk	0.89
Stand	0.89
Piloerection	*0.79*
