# Supplementary material for: Purr-ceiving feelings: domestic cats respond to intraspecific cues of emotion
Source: PeerJ. 2026 May 25;14:e21292. doi: 10.7717/peerj.21292 (PMC13218337; doi:10.7717/peerj.21292)
Supplement: Supplemental Information 8 [file peerj-14-21292-s008.pdf]

**Omitted behaviours/FACS**	**Cohen's κ**
Tail twitch	0.18
EAD101	0.08
EAD102	0.17
EAD104	0.14
EAD105	0.25
EAD106	0.01
AU200	0.29
AU201	0.28
AU5	-0.10
AU47	0.27
