# Supplementary material for: Purr-ceiving feelings: domestic cats respond to intraspecific cues of emotion
Source: PeerJ. 2026 May 25;14:e21292. doi: 10.7717/peerj.21292 (PMC13218337; doi:10.7717/peerj.21292)
Supplement: Supplemental Information 9 — Descriptions of the general behaviours were adapted from Stanton et al. (Stanton, Sullivan & Fazio, 2015), descriptions of the vocalizations were adapted from Tavernier et al. (Tavernier et al., 2020), and descriptions of FACS behaviours were taken from the CatFACS manual (freely available on www.CatFACS.com). [file peerj-14-21292-s009.pdf]

Behaviour	Description
Stimulus-directed	
Approach	Cat moves toward stimulus while looking at it.
Sniff	Cat sniffs stimulus by inhaling air through the nose from within 50 cm.
Static	
Lay	Cat's body is on the ground in a horizontal position on its side, back or belly.
Sit	Cat is in an upright position, with the hind legs flexed and resting on the ground, while front legs are extended and straight.
Stand	Cat is in an upright position and immobile, with all four paws on the ground and legs extended, supporting the body.
Body posture	
Crouch	Cat is alert and positions the body close to the ground, whereby all four legs are bent, and the belly is touching (or raised slightly off of) the ground.
Freeze	Cat suddenly becomes immobile, body is tense.
Tail position	
Slap	Cat quickly strikes its tail on the ground.
Swish	Cat violently swishes the tail.
Up	Cat holds the tail in an upright position. The tip may be slightly curved.
Wave	Cat slowly and gently waves the tail from side to side.
Vocalization	
Meow	The distinctive "meow" call that is typical of cat, the mouth is opened and closed gradually.
Purr	Continuous murmuring sound, produced during respiration with a closed mouth.
Vocal (other)	Any vocalization that cannot be categorized as a growl, hiss, meow, purr, trill, or yowl.
Other behaviours	
Body/head shake	Cat rotates its head, a body part or the entire body from side to side.
Groom	Cat cleans itself by licking, biting, or chewing the fur on its body. May include the licking of a front paw and wiping it over one's head.
Interaction owner	Cat seeks contact with owner by gazing, vocalizing while gazing/approaching or seeking physical contact (e.g. rubbing).
Interaction exp.	Cat seeks contact with experimenter by gazing, vocalizing while gazing/approaching or seeking physical contact (e.g. rubbing).
Piloerection	Cat raises the hairs on the nape of its neck, shoulder, back or tail, so that the fur is standing erect.
Skin twitch	Cat quickly twitches its skin near the base of the tail in a small amplitude movement.
Walk	Cat locomotes forward at a slow gait.

FACS action unit	Description
Eye blink (AU145)	The upper and lower eyelids move towards each other, eventually closing the eye(s). The eye(s) open(s) within half a second.
Nose lick (AD137)	The tongue is moved beyond the lips and in a dorsal movement, wiping the nose.
Pupil dilation (AD68)	Pupils increase in size.
