# Supplementary material for: Purr-ceiving feelings: domestic cats respond to intraspecific cues of emotion
Source: PeerJ. 2026 May 25;14:e21292. doi: 10.7717/peerj.21292 (PMC13218337; doi:10.7717/peerj.21292)
Supplement: Supplemental Information 10 — a Results from step 1 of the hurdle model (GLMM binomial), b Results from step 2 of the hurdle model (GLMM with gamma distribution). •p<0.1, *p<0.05, **p<0.01, ***p<0.001. [file peerj-14-21292-s010.pdf]

Behavioural measure (Visual condition)	Predictor	Effect modificator	ß	t	z	df	*p*	95% CI Lower	Upper
Attention									
Gaze duration	Valence [neg]	Activity [passive]	1.104	0.572	-	69.000	0.569	0.789	1.545
		Activity [active]	1.898	3.705	-	69.000	0.000***	2.653	1.355
	Valence x Activity		1.718	2.215	-	69.000	0.030*	1.068	2.763
Gaze frequency	Valence [neg]	Activity [passive]	0.761	-	-1.516	-	0.130	0.534	1.084
		Activity [active]	1.316	-	1.764	-	0.078 •	0.737	1.783
	Valence x Activity		1.729	-	2.299	-	0.022*	1.094	2.758
Stimulus interaction									
Interaction occurrence[a]	Valence [neg]	Activity [passive]	0.969	-	1.354	-	0.176	-0.399	2.447
		Activity [active]	1.024	-	1.386	-	0.166	-0.383	2.560
	Valence x Activity		0.055	-	0.055	-	0.956	-1.939	2.062
Interaction duration[b]	Valence [neg]	Activity [passive]	1.903	1.962	-	41.052	0.056 •	1.004	3.573
		Activity [active]	0.773	-0.895	-	39.543	0.376	1.342	0.441
	Valence x Activity		0.407	2.092	-	37.222	0.043*	0.177	0.932
Proximity									
Duration within 150 cm	Valence [neg]	Activity [passive]	1.086	0.240	-	69.000	0.811	0.555	2.125
		Activity [active]	1.111	0.305	-	69.000	0.761	0.568	2.174
	Valence x Activity		1.023	0.046	-	69.000	0.963	0.396	2.642
Frequency within 150 cm	Valence [neg]	Activity [passive]	1.128	-	0.548	-	0.583	0.733	1.736
		Activity [active]	0.870	-	-0.646	-	0.518	0.569	1.328
	Valence x Activity		0.771	-	-0.844	-	0.398	0.421	1.411
Duration within 50 cm[b]	Valence [neg]	Activity [passive]	1.488	1.231	-	42.368	0.225	0.797	2.774
		Activity [active]	1.429	1.280	-	39.909	0.208	0.834	2.445
	Valence x Activity		0.959	0.099	-	39.269	0.922	0.426	2.161

Frequency within 50 cm	Valence [neg]	Activity [passive]	1.750	-	1.546	-	0.122	0.861	3.557
		Activity [active]	1.105	-	0.316	-	0.752	0.594	2.058
	Valence x Activity		0.632	-	-0.956	-	0.339	0.869	1.621
Approach latency[b]	Valence [neg]	Activity [passive]	1.030	0.663	-	-	0.507	0.944	1.123
		Activity [active]	0.971	-0.663	-	-	0.507	0.891	1.059
	Valence x Activity		0.944	0.810	-	-	0.418	0.821	1.085
Out of sight occurrence[a]	Valence [neg]	Activity [passive]	1.159	-	1.269		0.205	-0.568	3.101
		Activity [active]	-1.440	-	-1.602		0.109	-3.376	0.239
	Valence x Activity		2.599	-	-1.959		0.050 •	0.149	5.470
Out of sight duration[b]	Valence [neg]	Activity [passive]	1.000	0.049	-	-	0.961	0.995	1.006
		Activity [active]	1.001	0.052	-	-	0.958	1.006	0.994
	Valence x Activity		1.000	0.007	-	-	0.995	0.992	1.008
Cluster 1	Valence [neg]	Activity [passive]	1.058	-	0.473	-	0.636	0.884	1.334
		Activity [active]	0.954	-	-0.408	-	0.684	0.759	1.198
	Valence x Activity		0.902	-	-0.623	-	0.533	0.651	1.249
Cluster 2	Valence [neg]	Activity [passive]	0.651	-	-1.767	-	0.077 •	0.405	1.048
		Activity [active]	1.737	-	2.711	-	0.007 **	1.165	2.589
	Valence x Activity		2.667	-	3.096	-	0.002**	1.433	4.964
Cluster 3	Valence [neg]	Activity [passive]	1.157	-	0.756	-	0.450	0.793	1.688
		Activity [active]	1.130	-	0.731	-	0.465	0.814	1.567
	Valence x Activity		0.977	-	-0.094	-	0.925	0.594	1.606
Cluster 4	Valence [neg]	Activity [passive]	0.951	-	-0.224	-	0.823	0.614	1.475
		Activity [active]	0.789	-	-0.968	-	0.333	0.489	1.274
	Valence x Activity		0.830	-	-0.563	-	0.574	0.434	1.588

Behavioural measure (Auditory condition)	Predictor	Effect modificator	ß	t	z	df	*p*	95% CI Lower	Upper
Attention									
Gaze duration	Valence [neg]	Activity [passive]	0.624	-2.195	-	69.000	0.032 *	0.411	0.948
		Activity [active]	0.889	-0.546	-	69.000	0.587	0.586	1.350
	Valence x Activity		1.424	1.166	-	69.000	0.248	0.790	2.570
Gaze frequency	Valence [neg]	Activity [passive]	0.747	-	-1.818	-	0.069 •	0.546	1.023
		Activity [active]	0.955	-	-0.305	-	0.760	0.708	1.287
	Valence x Activity		1.277	-	1.106	-	0.269	0.828	1.971
Stimulus interaction									
Interaction occurrence[a]	Valence [neg]	Activity [passive]	-0.847	-	-1.091	-	0.275	-2.512	0.627
		Activity [active]	-0.274	-	-0.369	-	0.712	-1.796	1.191
	Valence x Activity		0.573	-	0.533	-	0.594	-1.531	2.749
Interaction duration[b]	Valence [neg]	Activity [passive]	1.514	0.856	-	4.826	0.432	0.586	3.911
		Activity [active]	1.459	0.714	-	10.624	0.490	0.517	4.115
	Valence x Activity		0.964	0.054	-	5.434	0.959	0.255	3.641
Proximity									
Duration within 150 cm[b]	Valence [neg]	Activity [passive]	1.201	0.468	-	56.677	0.642	0.564	2.567
		Activity [active]	1.329	0.816	-	52.509	0.418	0.677	2.616
	Valence x Activity		1.107	0.193	-	55.444	0.848	0.400	3.058
Frequency within 150 cm	Valence [neg]	Activity [passive]	0.774	-	-0.941	-	0.347	0.454	1.319
		Activity [active]	1.143	-	0.516	-	0.606	0.688	1.898
	Valence x Activity		1.476	-	1.038	-	0.299	0.707	3.081
Duration within 50 cm[b]	Valence [neg]	Activity [passive]	0.956	-0.068	-	19.008	0.947	0.277	3.327
		Activity [active]	0.930	-0.114	-	17.319	0.911	0.275	3.334
	Valence x Activity		0.973	-0.031	-	15.821	0.976	0.181	5.602

Frequency within 50 cm	Valence [neg]	Activity [passive]	0.455	-	-1.462	-	0.144	0.158	1.308
		Activity [active]	0.625	-	-0.824	-	0.410	0.204	1.910
	Valence x Activity		1.375	-	0.406	-	0.685	0.295	6.402
Approach latency[b]	Valence [neg]	Activity [passive]	1.037	0.071	-	20.079	0.944	0.405	2.651
		Activity [active]	0.724	-0.667	-	18.956	0.513	0.289	1.792
	Valence x Activity		0.698	0.521	-	17.890	0.609	0.189	2.529
Out of sight occurrence[a]	Valence [neg]	Activity [passive]	0.000	-	0.000	-	1.000	-1.307	1.307
		Activity [active]	0.000	-	0.000	-	1.000	-1.311	1.311
	Valence x Activity		0.000	-	0.000	-	1.000	-1.851	1.851
Out of sight duration[b]	Valence [neg]	Activity [passive]	1.009	1.692	-	-	0.091 •	0.999	1.020
		Activity [active]	1.000	-0.014	-	-	0.989	0.991	1.009
	Valence x Activity		0.991	1.308	-	-	0.191	0.977	1.005
Cluster 1	Valence [neg]	Activity [passive]	1.038	-	0.333	-	0.739	0.835	1.290
		Activity [active]	0.988	-	-0.111	-	0.912	0.795	1.227
	Valence x Activity		0.952	-	-0.314	-	0.753	0.700	1.295
Cluster 2a	Valence [neg]	Activity [passive]	0.681	-	-1.532	-	0.126	0.417	1.113
		Activity [active]	0.944	-	-0.270	-	0.787	0.623	1.432
	Valence x Activity		0.722	-	0.989	-	0.323	0.378	1.377
Cluster 2b	Valence [neg]	Activity [passive]	1.261	-	1.174	-	0.240	0.856	1.857
		Activity [active]	1.055	-	0.282	-	0.778	0.729	1.525
	Valence x Activity		0.836	-	-0.655	-	0.512	0.490	1.428
